# Comprehensive genomic surveillance reveals transmission profiles of extensively drug-resistant tuberculosis cases in Pará, Brazil

**DOI:** 10.3389/fmicb.2024.1514862

**Published:** 2025-01-22

**Authors:** Davi Josué Marcon, Abhinav Sharma, Alex Brito Souza, Rafaella Bonfim Barros, Valnete das Graças Dantas Andrade, Ricardo José de Paula Souza Guimarães, Luana Nepomuceno Gondim Lima, Lúcia Helena Martins Tavares Monteiro, Ana Judith Pires Garcia Quaresma, Layana Rufino Ribeiro, Philip Noel Suffys, Robin Mark Warren, Carlos Augusto Abreu Alberio, Karla Valéria Batista Lima, Emilyn Costa Conceição

**Affiliations:** ^1^Programa de Pós-Graduação em Biologia Parasitária na Amazônia, Belém, Brazil; ^2^Seção de Bacteriologia do Instituto Evandro Chagas, Ananindeua, Pará, Brazil; ^3^South African Medical Research Council Centre for Tuberculosis Research, Division of Molecular Biology and Human Genetics, Faculty of Medicine and Health Sciences, Stellenbosch University, Cape Town, South Africa; ^4^Laboratório Central do Estado do Pará, Belém, Pará, Brazil; ^5^Seção de Epidemiologia do Instituto Evandro Chagas, Ananindeua, Pará, Brazil; ^6^Secretaria de Estado da Saúde do Pará, Belém, Pará, Brazil; ^7^Programa de Pós-graduação em Epidemiologia e Vigilância em Saúde, Ananindeua, Brazil; ^8^Laboratório de Biologia Molecular Aplicada a Micobactérias, Instituto Oswaldo Cruz, Fundação Oswaldo Cruz, Rio de Janeiro, Brazil; ^9^Hospital Universitário João de Barros Barreto, Ambulatório de Tuberculose Multiressistente, Universidade Federal do Pará, Belém, Pará, Brazil

**Keywords:** bedaquiline, drug-resistant tuberculosis, extensively drug-resistant tuberculosis, genomic surveillance, whole-genome sequencing

## Abstract

Bedaquiline, an antimicrobial used to treat drug-resistant tuberculosis (DR-TB), was introduced in Brazil in October 2021. Monitoring the emergence and transmission of DR-TB is crucial for implementing public health to control the spread of DR strains of *Mycobacterium tuberculosis*. To measure its impact on the multi-drug treatment scheme in the state of Pará, we aimed to conduct genomic surveillance of DR-TB after bedaquiline was introduced in Brazil. Individuals treated for DR-TB between October 2021 and December 2022, in the reference hospital to treat DR-TB cases from the state of Pará, were included in the study. Clinical and bacteriological information was obtained from the National Laboratory Management Environment and the Special TB Treatment Information System. Genomic DNA was extracted from bacterial cultures performed at the Pará Central Laboratory (LACEN-PA). Whole-genome sequencing (WGS) was obtained using Illumina Nextera-XT and NextSeq 550 and genomes were analyzed using the MAGMA and TB-Profiler pipelines interpreted according to the World Health Organization (WHO) mutations catalog 2nd edition. Geoprocessing was performed based on the patient’s residences. Cutoffs of 5–12 single nucleotide polymorphisms (SNPs) were used for transmission analysis. From the 103 patients reported as DR-TB, viable cultures were obtained from 67. Forty isolates were selected randomly for WGS. Among these, a mixed infection of *M. tuberculosis* L1 and L4 and a co-infection of *M. tuberculosis* and *Mycobacterium chelonae* were observed. The genotypic drug susceptibility profile of TB stains (39/40) was as follows: sensitive (1/2, 5%), rifampicin mono-resistant (RR) (4/10%), isoniazid mono-resistant (1/2%), multidrug-resistant (MDR) (21/52%), extensively drug-resistant (XDR) (3/7%), pre-XDR (8/20%), and other (1/2%). Among the 38 isolates of *M. tuberculosis* strains without mixed infection, using a cutoff of 12 SNPs and suggestive of recent TB transmission, 14 (37%) were grouped into five clusters (C1–C5) and included RR (C5), MDR (C3, C4, C5), pre-XDR, and XDR (C2) strains. We recommend greater attention from the regional public health authorities to detect and track resistance to new drugs, especially in areas with pre-XDR and XDR cases. This is the first report on the detection and transmission of XDR-TB in Pará, Brazil, after the recent re-definition of XDR-TB by the WHO in 2021.

## 1 Introduction

Whole genome sequencing (WGS) is rapidly transforming tuberculosis (TB) surveillance, particularly in the context of new drugs like bedaquiline. According to the latest World Health Organization (WHO) definition of drug-resistant tuberculosis (DR-TB), there are different levels of resistance with epidemiological importance: TB only resistant to isoniazid (HR), TB only resistant to rifampicin (RR-TB), multidrug-resistant TB (MDR-TB, resistant at least to isoniazid and rifampicin), pre-extensively drug-resistant TB (pre-XDR-TB, resistant to rifampicin, isoniazid, and any fluoroquinolone), and extensively drug-resistant TB (XDR-TB, resistant to rifampicin, isoniazid, and any fluoroquinolone, and at least one of the newer drugs bedaquiline and linezolid) (WHO, 2021). Introduced in Brazil in 2021 for the treatment of RR, MDR, and pre-XDR TB (Brasil, 2020), the addition of bedaquiline marked a significant advancement in therapy. However, cases of bedaquiline resistance have already emerged in countries such as China, South Africa, Moldova, and Indonesia, highlighting the need for close monitoring when using this and other new drugs (Chesov et al., 2021; Tong et al., 2023; Rukmana et al., 2024).

Traditional methods for diagnosing *Mycobacterium tuberculosis* drug resistance can be slow and limited, especially for new drugs. In this context, WGS offers a significant advantage by providing a comprehensive overview of the genetic profile of a particular TB strain (Cohen et al., 2019; Sonnenkalb et al., 2023). This allows researchers and public health authorities to identify existing and undescribed mutations associated with DR-TB (WHO, 2023).

Furthermore, WGS can reveal TB transmission patterns, along with surveillance of the development and spread of DR-TB strains (Satapathy et al., 2024). Combined with conventional epidemiologic and clinical data, this information can guide targeted public health interventions, ensuring more precise and effective approaches. Additionally, WGS can inform treatment rescue strategies in cases of observed resistance or treatment failure, especially in high-burden TB countries, such as Brazil, which is relevant considering a recent observation that XDR-TB strains are possibly circulating in the state of São Paulo already^1^.

In 2023, Pará, a state in Brazil’s Northern region, ranked sixth nationally for TB incidence (49.4 cases per 1,000 inhabitants), with a concerning rise in DR-TB in recent years (da Brasil, 2024). Given the context of the new drug introduction in November 2021 for MDR-TB and later for RR-TB, this study aimed to (i) conduct genomic surveillance using WGS on early cases of RR/MDR-TB from Pará treated with bedaquiline and (ii) compare WGS data with the current standard of care (SOC) approach in Brazil, which includes GeneXpert, phenotypic drug susceptibility test (pDST), and line-probe assays (LPA).

## 2 Materials and methods

### 2.1 Ethics aspect

This study was approved by the Ethics and Research Committee of the Evandro Chagas Institute (CEP/IEC) CAAE 0821119.4.0000.0019, approval number: 3.950.56.

### 2.2 Study design and inclusion/exclusion criteria

**Figure 1:**
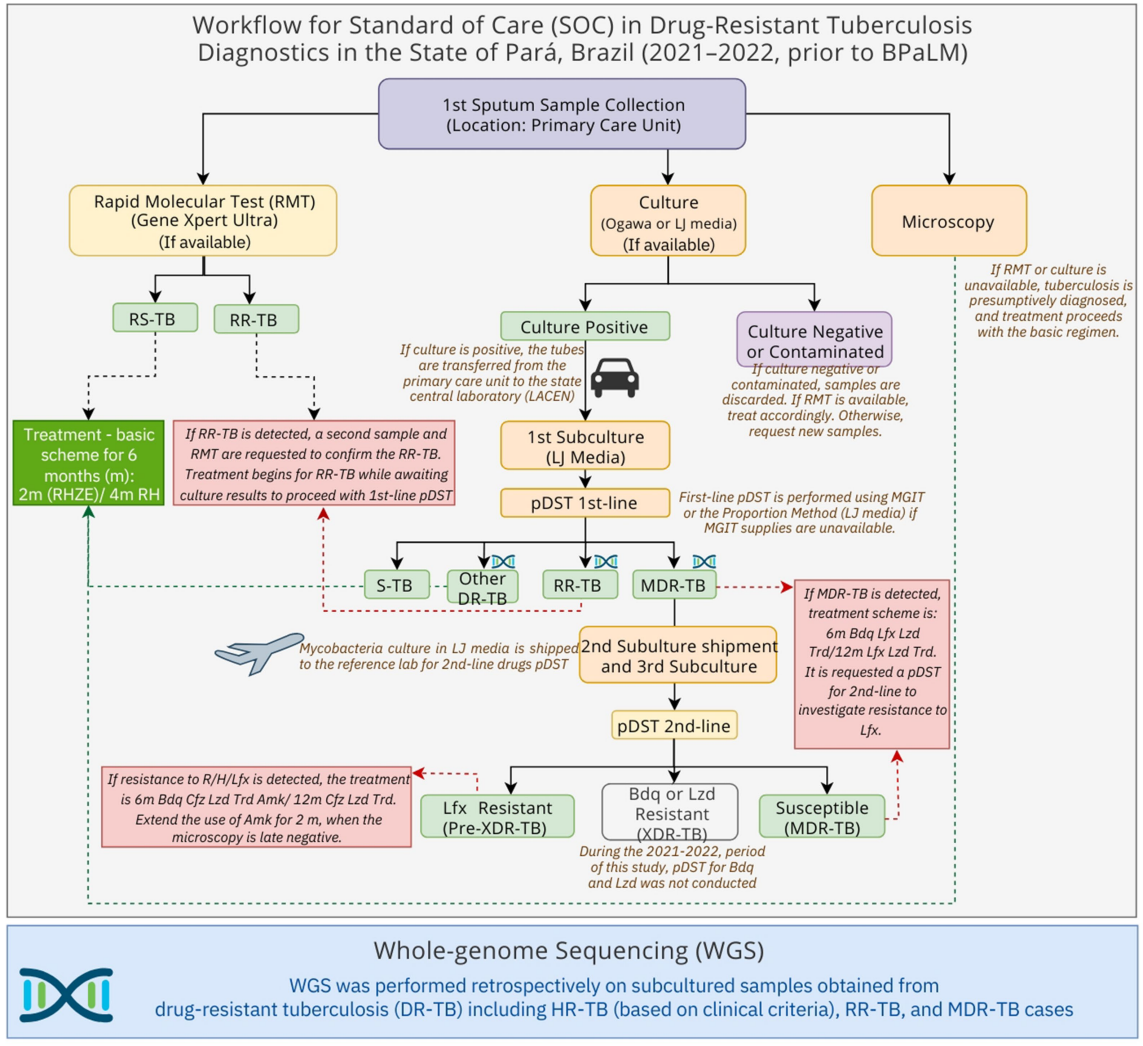
Schematic overview of the standard-of-care tuberculosis diagnostic and treatment workflow in Pará State, Brazil (November 2021–December 2022), and sample selection for whole-genome sequencing (WGS). Drug-resistant tuberculosis (DR-TB), Rifampicin-Resistant tuberculosis (RR-TB), Multidrug-resistant tuberculosis (MDR-TB), Phenotypic drug-susceptibility test (pDST), 1st (first)-line drugs: rifampicin, isoniazid, pyrazinamide and ethambutol, 2nd (second)-line drugs: levofloxacin, amikacin, bedaquiline, linezolid. Bedaquiline (Bdq), Linezolid (Lzd), Levofloxacin (Lfx), Susceptible tuberculosis (S-TB), months (m), Rifampicin (R), H (Isoniazid), Pyrazinamide (Z), Ethambutol (E).

This retrospective, cross-sectional, and observational study included baseline sputum samples from individuals with pulmonary TB, broadly classified as DR-TB (encompassing RR-TB, isoniazid monoresistant [HR]-TB, and MDR-TB), according to the SOC routine diagnostics (Figure 1). All patients were treated with a bedaquiline-based regimen.

All cryopreserved culture samples, derived from sputum samples that were collected from November 2021 to December 2022, were included in this study following the inclusion criteria: (i) pulmonary TB; (ii) treated or undergoing treatment for RR-TB, HR-TB (if in retreatment or with commodities), or MDR-TB; (iii) residents of the state of Pará at the time of TB diagnosis. The exclusion criteria were as follows: (i) affected exclusively by non-tuberculous mycobacteria (NTM); (ii) *M. tuberculosis* did not remain viable for culture or subculture.

### 2.3 Drug susceptibility testing and treatment

When a sample tested positive for RR-TB using the Xpert MTB/RIF Ultra assay (Cepheid, Sunnyvale, United States), a second sample was collected to confirm the resistance. This second sputum sample was processed and decontaminated using the *N*-acetyl-l-cysteine sodium hydroxide (NALC/NaOH) protocol, followed by culturing in Löwenstein–Jensen (L-J) medium at the Central Laboratory of Pará (LACEN-PA) (Brasil, 2022).

Further investigation was conducted through first-line pDST for isoniazid, rifampicin, ethambutol, and streptomycin, as performed at LACEN-PA (Brasil, 2022), using either automated mycobacteria growth indicator tubes (MGIT) (Becton, Dickinson and Company, Sparks, USA) or the proportion method. An aliquot of each sample was cryopreserved at −80°C for traceability.

For cases confirmed as MDR-TB, the samples were transported to the National Reference Mycobacteria Laboratory at the Professor Hélio Fraga Reference Center—Fiocruz (Jacarepaguá), Rio de Janeiro. Second-line pDST for fluoroquinolones and aminoglycosides was performed using either the MGIT system or the line probe assay MTBDR-sl (Hain Lifescience, Nehren, Germany). First-line pDST was also conducted on first sputum samples and compared to the results of the RMT when the test was available (Figure 1).

All individuals with samples classified as RR-TB by either Xpert MTB/RIF Ultra and/or pDST for first-line drugs were referred to the University Hospital João de Barros Barreto (HUJBB) at the Federal University of Pará (UFPA), a reference hospital for the treatment of DR-TB in the state of Pará. The treatment scheme followed the Brazilian Ministry of Health guidelines (Brasil, 2019, 2020).

### 2.4 Data acquisition

The clinical, epidemiological, sociodemographic, and bacteriological data were obtained through partnerships with the LACEN-PA and HUJBB/UFPA. These institutions have access to data from the national automated systems from the Laboratory Environment Manager (GAL)^2^ and the Tuberculosis Special Treatment Information System (SITETB)^3^. Bacteriological data were obtained from GAL, including first and second-line pDST. The laboratory results were obtained from standard procedures established by the Brazilian Ministry of Health (Brasil, 2022) used to determine the drug susceptibility pattern in the SOC. The final data collection date was done on 25 July 2024.

### 2.5 DNA extraction and whole-genome sequencing

Cryopreserved bacteria were subcultured in L-J media, followed by a heat inactivation, and three loops of 10 μL bacterial biomass were resuspended in 200 μL of water, and incubated at 80°C for 1 h using a Vulcan thermoblock (Biancodent, Araucaria, Brazil). The DNA extraction was performed using the MagMAX™ Viral/Pathogen Ultra Nucleic Acid Isolation Kit (Thermo Fisher, Waltham, United States) and purified with Ampure XP magnetic beads (Beckman Coulter, Brea, United States). The DNA quality control (QC) was performed by visualization of the DNA integrity through electrophoresis in 1.5% agarose gel and quantification using the double-strand (ds) DNA High Sensitivity (HS) Qubit Kit (Thermo Fisher, Waltham, United States).

We randomly selected 40 samples for WGS using the online tool https://www.randomizer.org/. These selections were made independently of laboratory results or patient clinical data. The genomic library preparation was performed using Nextera XT Library Preparation Kit (Illumina, San Diego, United States) followed by quality-controlled using Agilent Bioanalyzer 2,100 High Sensitivity Double Strand DNA (Thermo Fisher, Waltham, United States) and quantification using Qubit ds-HS kit. The genomic libraries were pooled and sequenced using the Illumina NextSeq 550 system, Mid-Output Kit, 2 × 150 bp, paired-end, with an approximate 100 times coverage.

### 2.6 Genomic analysis and geoprocessing

The FASTQ files were processed using the MAGMA pipeline v.2.0.0, covering steps from quality control to drug resistance prediction and phylogenetic analysis. Detailed descriptions of the base call and mapping quality parameters can be found on the MAGMA GitHub page^4^ and in the pipeline publications (Heupink et al., 2021, 2023). This pipeline includes representative samples from the 10 human-adapted lineages (L1–L10), as well as the outgroup *Mycobacterium canettii*, facilitating the positioning of study samples for phylogenetic inference analysis.

**Figure 2:**
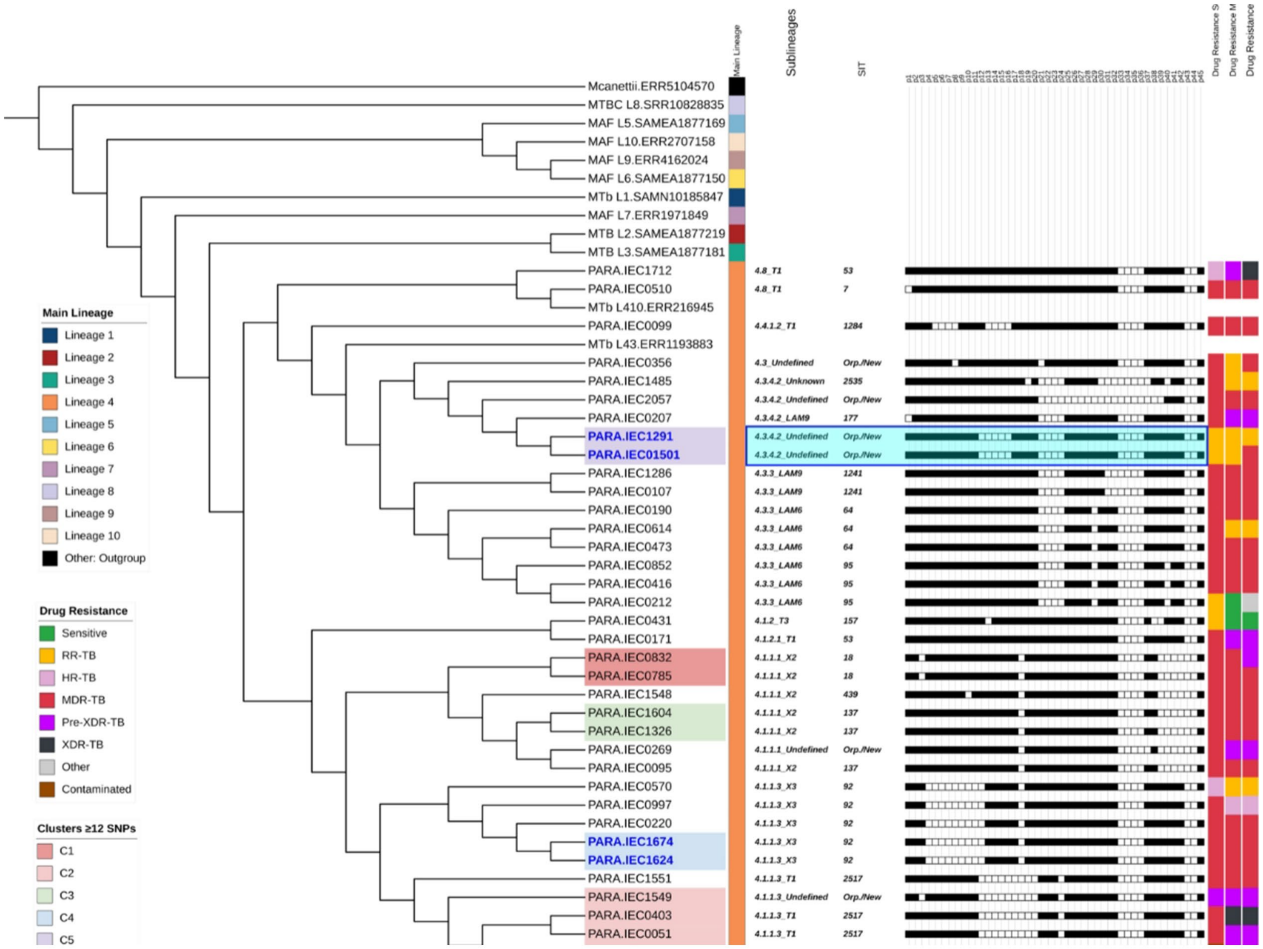
Phylogenetic tree based on Whole-Genome Sequencing (WGS), illustrating strain correlations and their respective profiles added as tree annotations after each sample name within the following order: (1) predicted lineages and sublineages, (2) Shared International Typing (SIT), and (3) spoligotyping profiles for the 43 spacers. The figure also presents drug susceptibility status based on (4) standard of care (SOC), and WGS (5) major variants, and (6) WGS (combined major and minor variants combined). The highlighted clades represent five clusters (C1–C5), identified using a ≤12 Single Nucleotide Polymorphism (SNP) threshold, while sample names highlighted in deep blue indicate strains with distances of ≤5 SNPs. The light blue highlight of C5 spoligotype pattern demonstrates a potential new SIT.

The phylogenetic analysis was performed as previously described (Heupink et al., 2021, 2023). Briefly, for the phylogenetic and cluster analyses, we concatenated filtered SNPs into a multi-FASTA file. We excluded sites represented by less than 95% of samples, common drug-resistance variants, and rRNA gene variants to avoid phylogenetic bias. SNP distances between sample pairs were calculated, both including and excluding complex regions of the *M. tuberculosis* genome. Cluster identification was performed using ClusterPicker with SNP cutoffs of 5 bp and 12 bp. IQtree was used to determine the most likely substitution model and compute the maximum-likelihood tree, which was visualized with iTOL v6^5^ (Figure 2), incorporating drug resistance and lineage markers. The standard MAGMA parameters for establishing cutoffs for median coverage (10), breadth of coverage (0.9), NTM fraction (0.2), site representation (0.95), and strand bias (0.05) were maintained. To avoid the inclusion of a false-positive sublineage 4.9, the relative abundance cutoff was lowered from 0.8 to 0.6 before proceeding with further analysis.

In addition to the lineage prediction, TB-Profiler v6.3.0 (Verboven et al., 2022) was used for genotypic DST (gDST) profiling as part of the MAGMA pipeline, which was interpreted based on the WHO catalog of mutations in *M. tuberculosis* complex (MTBC) and their association with drug resistance, second edition (WHO, 2023). This catalog determines confidence of variants in grades: (1) associated with resistance, (2) associated with resistance—interim, (3) uncertain significance, (4) not associated with resistance—interim, and (5) not associated with resistance. Only variants detected by MAGMA with either high or low frequency (detection limit ≥3%) classified by WHO as associated with resistance, including the interim, were used to classify samples as *M. tuberculosis* drug-resistant. The high-frequency variants (major) were obtained from XBS variant calling (Heupink et al., 2021) and low-frequency variants (minor) using lofreq variant caller optimized with higher sensitivity to the detection of variants occurring in <0.05% of a population (Wilm et al., 2012).

The transmission network analysis was performed using a ≤5 and ≤12 single nucleotide polymorphisms (SNPs) cutoff, which was calculated by MAGMA and submitted to the transcluster R package (Stimson et al., 2019) for prediction based on the first TB symptoms date obtained from SITE-TB. The spoligotype profile was predicted using SpoTyping v2.0^6^ and submitted to the SITVIT2 database^7^ for Spoligotyping International Type (SIT) determination. The spatial distribution was performed using ArcGIS software^8^ considering the patient’s residence address.

## 3 Results

From November 2021 to December 2022, 103 individuals diagnosed with RR-TB initiated treatment that included bedaquiline. However, among these, 36 patients were excluded from this study due to the following reasons: 21 (20.39%) samples were not localized in the cryopreservation isolate collection, 14 (13.59%) samples did not yield viable bacilli upon culturing and individuals had been treated based on Xpert MTB/RIF Ultra results, and one sample (0.97%) was negative for *M. tuberculosis* with a presumptive diagnosis of NTM. Of the remaining 67 samples, 40 were randomly selected for WGS. All patients included in the present study ended the treatments in 1 year and 6 months.

### 3.1 Lineage description

From 40 samples, 39 were predicted as members of MTBC, sample IEC1387 was identified as a mixed infection with 98% mapped reads to *M. chelonae-abscessus* complex species and 0.4% of reads aligned to the reference genome (*M. tuberculosis*, H37Rv), based on that, it was analyzed separately on downstream analysis. Sample IEC2061 was identified as a polyclonal infection with mixed lineage caused by lineages 4.3.3 and 1.2.2.2 with frequencies of 46 and 50%, respectively. The 38 remaining samples were classified as lineage 4, with clades 4.1.1.3 (12/38; 31%), 4.3.3 (8/38; 21%), 4.1.1.1 (7/38; 18%), 4.3.4.2 (5/38; 13%), 4.8 (2/38; 5%) and one representative from each of the clades 4.1.2, 4.1.2.1, 4.3, and 4.4.1.2.

Among the 38 samples, spoligotyping identified six sublineages: T1 (10/38; 26%), LAM6 (6/38; 16%), X2 (6/38; 16%), X3 (5/38; 13%), LAM9 (3/38; 8%), and T3 (1/38; 2.63%). The remaining samples were classified as “unknown” or “undefined” sublineages. The most frequent spoligotype SIT were 2,517 (6/38; 16%), 92 (5/38; 13%), 64 (3/38; 7%), 95 (3/38; 7%), and 137 (3/38; 7%) (Supplementary Table S1). Six samples (16%) were classified either as new or orphan spoligotypes due to the lack of matches with the SITVIT2 database. The two samples (IEC1291 and IEC01501) were predicted as lineage 4.3.4.2 and had a single SNP difference and spoligotype pattern suggestive of being of a new sublineage. The phylogenetic tree (Figure 2) provides a detailed overview of the lineages, sublineages, spoligotyping, clustering, and drug resistance results.

### 3.2 Drug resistance descriptive analysis

**Figure 3:**
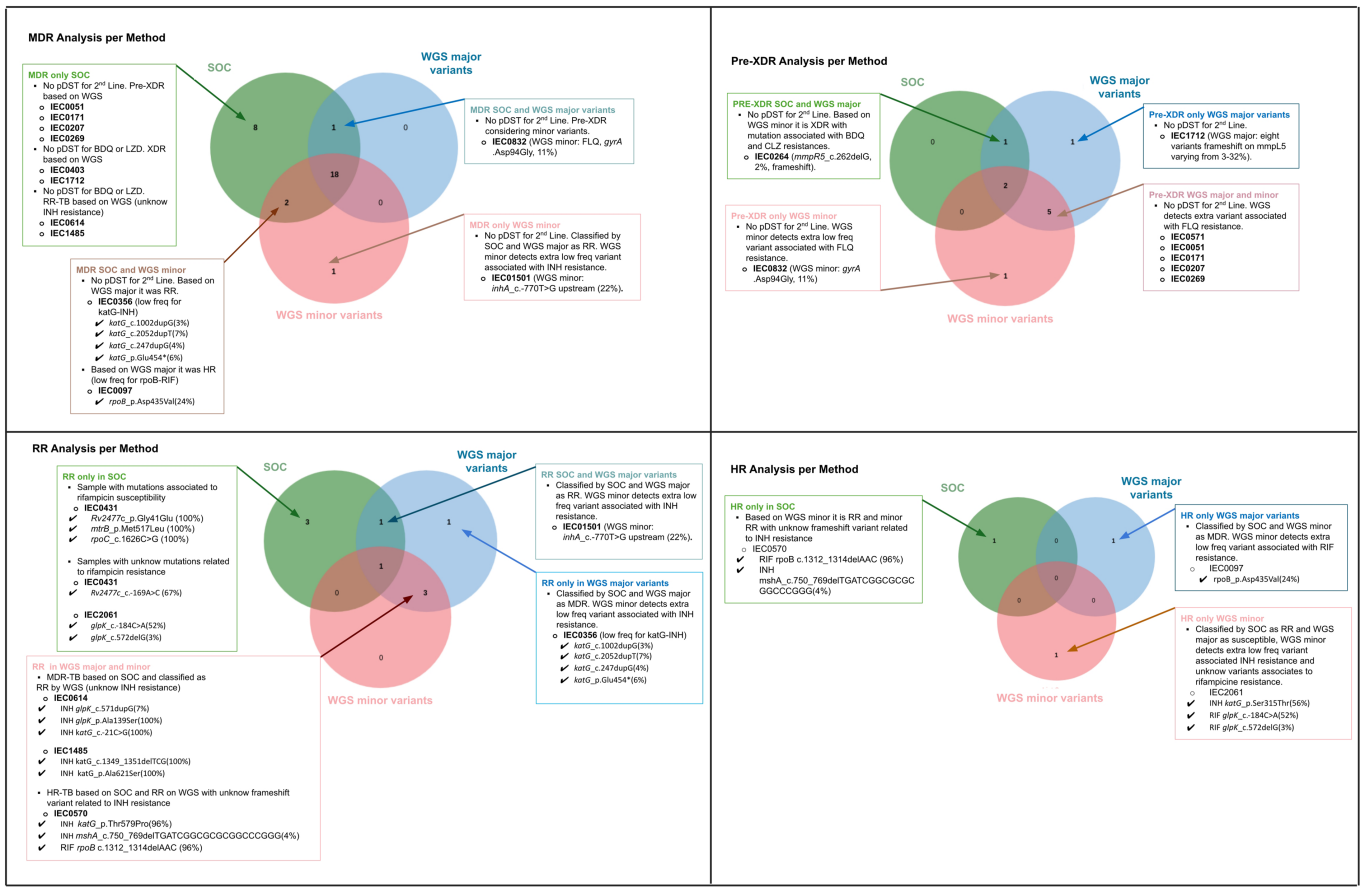
Comparative analysis of drug resistance profiles based on Standard of Care (SOC) and Whole-genome Sequencing (WGS), considering major and minor variants for each classification: multidrug-resistant (MDR), pre-extensively drug-resistant (Pre-XDR), rifampicin-resistant only (RR), and isoniazid-resistant only (HR). For samples with discordance across the three approaches, detailed explanations are provided for the discrepancies.

Figure 3 presents a comparison of the drug susceptibility profiles of the 39 *M. tuberculosis* strains, as determined by SOC and WGS (including major and minor variants). The figure highlights the concordances and discordances between the results obtained using these two approaches for MDR, pre-XDR, RR, and HR classifications. Other information regarding major and minor variants associated with resistance is available in Supplementary Table S2.

Based on the SOC results obtained from the 39 *M. tuberculosis * strains, the susceptibility profile for first-line antibiotics was as follows: 31 were MDR, five were RR, two were HR, and one sample was inconclusive due to culture contamination in both MGIT (SIRE Kit) and LJ (Proportion Method) pDST.

Based on the genotypic WGS profile, rifampicin resistance was associated with several *rpoB* variants: p.Asp435Val (3/35 8%), p.Gln432Pro (2/35 6%), p.His445Leu (1/35 3%), p.Leu430Pro (1/35 3%), and p.Ser450Leu (23/35 66%). While isoniazid resistance was associated with the high-frequency mutations *inhA*_c.-777C > T (4/31 12, 90%) and *katG*_p.Ser315Thr (26/31 84%). Fluoroquinolone resistance was associated with *gyrA*_p.Ala90Val (1/11, 9%), *gyrA*_p.Asp94Ala (1/11, 9%), *gyrA*_p.Asp94Asn (6/11, 54%), *gyrA*_p.Asp94Gly (3/11, 27%), ethambutol resistance was associated with mutations *embB*_p.Met306Ile (9/14, 64%) and *embB*_p.Gly406Ser (5/14, 36%). Pyrazinamide resistance was linked to multiple pncA mutations, including *pncA*_c.464dupT (7/21, 33%), *pncA*_p.Gln122* (3/21, 14%), pncA c.-11A > G (2/21, 9%), and *pncA*_p.Val128Gly (2/21, 9%). Streptomycin resistance was identified with *gid*_c.311delT (3/7, 57%), *rpsL*_p.Lys88Arg (2/7, 43%), *gid*_c.101dupG (1/7, 14%) and *gid*_c.102delG (1/7, 14%). We did not observe resistance associated with amikacin, kanamycin, and capreomycin.

We detected mutation in genes associated with resistance to new drugs such as bedaquiline, linezolid, clofazimine, delamanid, and pretomanid. For linezolid, the *rplC* p.Cys154Arg (100%) mutation was observed in sample IEC0403. Delamanid and pretomanid resistance was associated with the low-frequency mutations on genes *fbiB* and *fbiC* (*fbiB* c.83_105delATCTGAGCGCCGCCGTCGCCGCG *fbiB* c.381delC and *fbiC* p.Glu670*) being all of them with 2% frequency in samples such as IEC1549, IEC0852, IEC1674, and IEC0264.

**Figure 4:**
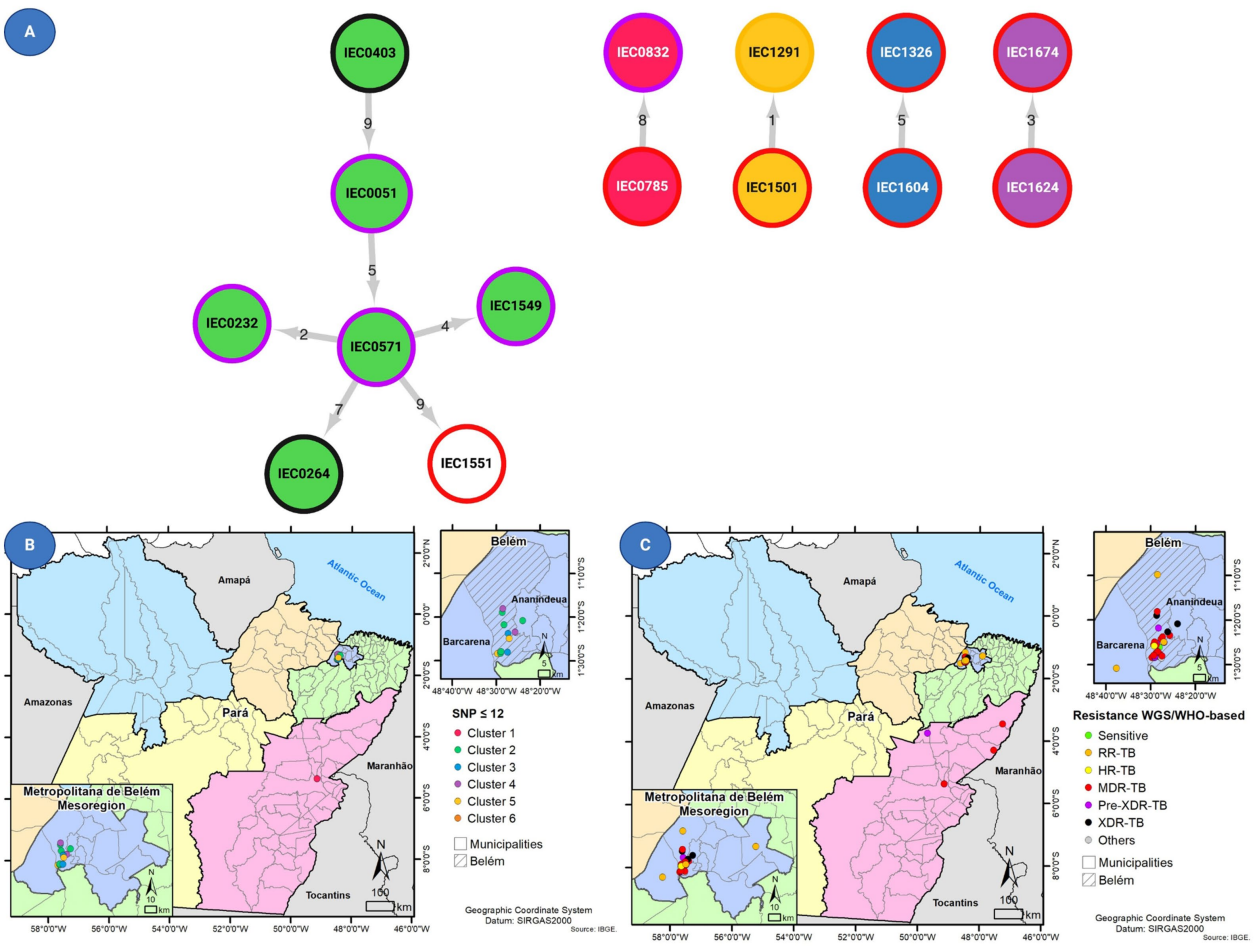
**(A)** Transmission network prediction with circle internal colors based on clusters and border color representing drug resistance status based on Whole-genome sequencing (WGS). In addition, the figure shows the geographical distribution of 14 patients with *Mycobacterium tuberculosis* isolates with **(B)** clustered genomes as defined using MAGMA and of 14 patients with isolates and **(C)** drug susceptibility profiling for all 39 tuberculosis cases.

Bedaquiline and clofazimine resistance was associated with different low-frequency variants (<40%). For bedaquiline, the detected mutations were *mmpR5* c.262delG (3%) in sample IEC0264, *mmpR5* c.386delC (2%) in sample IEC1485, and multiple mutations in *mmpR5* in sample IEC1712, including c.144dupC (32%), c.141_142dupTC (17%), c.424dupC (7%), c.321dupC (10%), c.139dupG (6%), c.140dupA (3%), c.270dupC (3%), and c.320_321dupGC (3%). The detailed clinical and genomic information about the three cases with bedaquiline resistance detected based on WGS are described in Table 1. When comparing the SOC results to WGS data for the 39 strains, 22 (56.41%) showed agreement using both WGS high-frequency variant and low-frequency detection analysis. Considering only high-frequency variants, 24 (61.54%) samples were concordant. For concordance based solely on lower frequency analysis, 23 (58.97%) agreed (Figure 4).

**Table 1:** Clinical and genomic data from three *Mycobacterium tuberculosis* strains exhibiting low-frequency mutations related to possible bedaquiline resistance, as determined by Whole-Genome Sequencing (WGS).

Sample ID		IEC0264	IEC1485	IEC1712
Lineage		4.1.1.3 (T1, SIT* 2517)	4.3.4.2 (U, SIT* 2535)	4.8 (T1, SIT* 53)
Type of diagnosis		New case	Return after lost to follow-up	Return after lost to follow-up
Treatment comments		Bedaquiline was removed from treatment because of adverse effects	–	Irregularity
Outcome		Lost to follow-up	Cured	Dead
Current Standard of Diagnostic		Pre-XDR-TB	MDR-TB	MDR-TB
WGS (considering major variants)		Pre-XDR-TB	MDR-TB	Pre-XDR-TB
WGS (considering minor variants)		XDR-TB	MDR-TB	XDR-TB
WGS final interpretation		XDR-TB (bedaquiline resistance detection in 3% of variants detection)	MDR-TB (bedaquiline resistance detection in 2% of variants detection)	XDR-TB (bedaquiline resistance detection at least 32% of variants detection)
Major variants associated with the following anti-TB drugs	Rifampicin	*rpoB* p.Ser450Leu (100%)	*rpoB p.Ser450Leu* (100%)	*rpoB p.Leu430Pro* (100%)
	Isoniazid	*katG* p.Ser315Thr (99%)	Frameshift mutations *katG_c.1349_1351delTCG* (100%) and *katG_p.Ala621Ser* (100%) – unknown relevance^#^	*katG_c.-343G > T* (3%), *katG_c.-697 T > A* (6%), *katG_p.Gln295Pro* (100%) – unknown relevance^#^
	Ethambutol	*embB* p.Gly406Ser (100%)	–	–
	Pyrazinamide	*pncA* c.464dupT (100%)	–	*pncA* p.Val139Gly (100%)
	Fluoroquinolone	*gyrA* p.Asp94Asn (100%)	–	*gyrA* p.Asp94Ala (100%)
	Ethionamide	*ethA* c.935dupT (100%)	–	–
Minor variants associated with the following new anti-TB drugs	Bedaquiline and clofazimine	mmpR5 c.262delG (3%)	*mmpR5* c.386delC (2%)	*mmpR5* [c.139dupG (6%), c.140dupA (3%), c.141_142dupTC (17%), c.144dupC (32%), c.270dupC (3%), c.320_321dupGC (3%), c.321dupC (10%), c.424dupC (7%)]
	Delamanid and pretomanid	*fbiB* c.83_105delATCTGAGCGCCGCCGTCGCCGCG (2%)	–	–

*Spoligotyping International Type (SIT).

^#Mutations with unknown resistance association by WHO Mutations Catalog, thus they are not considered for the status interpretation as isoniazid resistance.^

A total of 17 (41.02%) discordances were identified between SOC and WGS results considering both high-frequency and low-frequency variants. Among these, five (31.25%) were attributed to the absence of second-line testing in the SOC and isolates classified as MDR by pDST were reclassified as pre-XDR or XDR by gDST. The remaining 11 (68.75%) discrepancies were linked to lower frequency variants involving rifampicin, isoniazid, or fluoroquinolones (*n* = 4; 25%). Additionally, pre-XDR strains were reclassified as XDR due to the detection of mutations associated with resistance to new drugs (*n* = 2; 5%). Other discrepancies included unknown mutations related to isoniazid resistance (*n* = 2; 5%), inconclusive phenotypic tests (*n* = 1; 2%), one sample (*n* = 1; 2%) with discordant results due to inconclusive SOC, and one sample (2.5%) showing phenotypic RR while presenting a genomic variant associated with rifampicin susceptibility.

The mutations with unknown status of resistance to isoniazid were observed in three strains phenotypically resistant to isoniazid (IEC1485, IEC0614, and IEC1712). The mutations and frequency were *glpK*_p.Ala139Ser (100%), *katG*_c.-21C > G (100%), *katG*_c.-683 T > A (7%), *glpK*_c.571dupG (7%) in strain IEC0614, *katG*_c.1349_1351delTCG (100%), *katG*_p.Ala621Ser (100%) in sample 1,485, and *Rv2752c*_p.Leu333Pro (100%), *Rv2752c*_p.Thr329Pro (100%), *ahpC*_c.-52C > T (100%), *katG*_c.-343G > T (3%), *katG*_c.-697 T > A (6%), *katG*_p.Gln295Pro (100%), *dnaA*_p.Pro124Leu (100%), and *Rv1258c*_c.148dupG (1%) in sample IEC1712.

### 3.3 Tuberculosis transmission analysis

Among the 39 *M. tuberculosis* strains, 14 (35.9%) belonged to five clusters (C1–C5) considering the ≤12 SNP difference cutoff, composed of one cluster with six isolates and four with two isolates each (Figure 2). When increasing stringency for cluster definition to the ≤5 SNP threshold level, six strains remained clustered within three clusters considering ≤5 SNPs cutoff, which were C3 (containing two pre-XDR strains), C4 (with two MDR strains), and C5 (with one RR and one MDR strain).

Based on the transmission network analysis, the *M. tuberculosis* strain IEC0403 was predicted as the initial case of the main transmission chain (C2). Additionally, it was predicted as associated with the transmission event to IEC0051 distant by five SNP from sample IEC0571. Sample IEC0571 is associated with at least four transmission events, being two with close contact (≤5 SNP) and two distant contacts (≤12 SNP); those events were associated with the transmission of a pre-XDR isolate containing the same resistance-associated variants (*rpoB* p.Ser450Leu, *katG* p.Ser315Thr, and *gyrA* p.Asp94Asn), all of them occurring in the metropolitan region of Belém and identified as part of SIT 2517.

Strains within C4 shared less than five SNPs of distance, with only one shared resistance variant (*rpoB* p.Ser450Leu) associated with RR. Both strains were identified as monoresistant by SOC and WGS (major variants analysis). Similarly, C4 contained two MDR strains sharing *rpoB* (p.Asp435Val) and *katG* (p.Ser315Thr). One distant transmission event (C1) was observed outside the metropolitan region of Pará, with two cases from Marabá, which were identified by the same medical facility, sharing *rpoB* (p.Gln432Pro), *katG* (p.Ser315Thr), and *embB* (p.Met306Ile) mutations.

## 4 Discussion

This study presents a comprehensive analysis of the first published XDR and pre-XDR-TB cases identified in Pará, Brazil, using WGS, and based on the latest WHO drug resistance definition (WHO, 2021). By examining baseline samples from an initial cohort of RR and/or MDR-TB patients treated with a bedaquiline-based regimen, we were able to compare SOC data with WGS findings, investigate transmission dynamics, and characterize the genetic diversity of circulating *M. tuberculosis* strains.

Our genomic analysis revealed multiple transmission clusters, including strains with mixed infections, one involving different *M. tuberculosis* lineages and co-infection with NTM. This is consistent with reports from high-burden TB countries such as China and South Africa (Tong et al., 2023; Rukmana et al., 2024), where WGS has similarly revealed emerging resistance patterns. However, the identification of XDR-TB transmission in Pará, Brazil, represents a novel finding, underscoring the critical need for enhanced surveillance in the region.

In this study, all *M. tuberculosis* strains belonged to lineage 4, the most generalist and predominant lineage in Latin America. Within lineage 4, the spoligotype T1 (SIT 2517) was reported in a larger cluster of five strains carrying mutations associated with MDR-TB. The SIT 2517 spoligotype is uncommon, having been previously reported only in Pará, Brazil, and linked to MDR-TB in a cohort of strains isolated between 2000 and 2010 (Conceição et al., 2017, 2024). Our genomic surveillance data further support the association between SIT 2517 and MDR-TB, suggesting that this genotype has been circulating for at least 21 years, as observed in this study. Recent strains with additional mutations conferring fluoroquinolone resistance indicate the ongoing evolution of drug resistance toward pre-XDR status. In addition to the observed frequency of the rare sublineage T1 (SIT 2517), we identified potential new sublineages within clade lineage 4.2.4.2. These sublineages shared the octal spoligopattern “777,603,607,760,771” in two samples. This lineage is closely related to LAM9 SIT 177 (sample IEC0207), which differs in the presence of spacer 1 and the absence of spacers from 12 to 16. Future validation of this genotype signature and deposition of these novel spoligotypes in the SITVIT2 database will be crucial for their accurate classification and further understanding.

The state of Pará, Brazil, is the second largest state in the country in terms of area, and it covers approximately 1.2 million square kilometers (460,000 square miles) (IBGE, 2021). This vast territory is primarily made up of the Amazon rainforest. The majority of DR-TB cases in this study were found in Belém, but we observed evidence of transmission between Belém and Marabá, two cities located 440 km apart, as indicated by genetic similarities of less than 12 SNPs. In general, of the 39 *M. tuberculosis* strains, 20 strains were mainly divided into close clusters harboring 14 strains that formed five clusters with a ≤12 SNP difference and the stricter clusters harbored six strains of these being close clusters with a ≤5 SNP difference.

From a public health standpoint, priority should be given to closely monitoring individuals associated with the strain IEC0403, identified as the initial case in the main transmission chain. This strain was classified as XDR-TB with linezolid resistance conferred by the *rplC* p.Cys154Arg mutation at 100% frequency. Additionally, the strain IEC0571, linked to at least four transmission events, including two close contacts and two distant contacts, warrants increased surveillance. In particular, all patients infected by strains belonging to genotype SIT 2517 should be prioritized for contact tracing and further investigation. These measures align with the emerging field of Precision Public Health, which utilizes genomic, social, behavioral, and environmental data, and the application of artificial intelligence to TB control programs to enhance disease prevention and control (Roberts et al., 2024).

Strains IEC1387 and IEC2061 were identified as mixed infections through WGS, which are well documented in literature (Kargarpour Kamakoli et al., 2017; Pandey et al., 2020; Moreno-Molina et al., 2021) and can involve either multiple strains of *M. tuberculosis* (within the same or different lineages) or co-infection with NTM (Bjerrum et al., 2016; Huang et al., 2022). Distinguishing between these scenarios can be challenging, as standard diagnostic methods have limitations. Although the GenoType *Mycobacterium* CM (Hain LifeScience, Nehren, Germany) can sometimes identify mixed infections, WGS remains the most reliable method for precise differentiation, enabling the simultaneous detection and characterization of both NTM species and sublineages. Further strain analysis is essential to accurately determine its drug resistance profile.

During the treatment course of an individual from which sample IEC1387 was isolated, culture reversion after successive negative cultures was observed in treatment month 8, molecular identification-based Sanger sequencing of ribosomal RNA 16 s, genes *hsp65* and *rpoB* identified the culture isolate as part of *Mycobacterium abscessus–chelonae* complex. Based on GeneXpert initial result as rifampicin-resistant TB and the later isolation and identification of *M. abscessus–chelonae* complex, associated with the WGS analysis on which both *M. tuberculosis* and *M. chelonae* (part of *M. abscess–chelonae* complex) were identified, we were able to identify NTM-TB mixed infection. The responsible clinician team kept the ongoing treatment, and after the complete treatment with successive negative cultures and good clinical evolution, the final treatment outcome was successful. This success is aligned with MIC assays demonstrating the effectivity bedaquiline in *M. abscessus* strains (Brown-Elliott and Wallace, 2019), the effectivity of clofazimine *in vitro* (Hajikhani et al., 2021), and also with clinical study incorporating linezolid in *M. abscessus* treatment (Cheng et al., 2023). In this case, the standard regimen proposed for TB treatment composed of bedaquiline, linezolid, levofloxacin, and clofazimine, had good results.

When comparing the WGS gDST *versus* SOC we observed discrepancies, which were largely attributed to the absence of second-line drug testing in many cases for patients as part of SOC. Despite the recommendation from the Brazilian Health Ministry for the application of these tests should be conducted in all cases when MDR is detected in first-line tests (Brasil, 2021, 2022), there were operational challenges within the flow of the diagnostic algorithm protocol. For example, if the state-level laboratory (LACEN-PA) does not offer pDST for second-line drugs or the molecular gDST test LPA (MTBDRsl), specimens should be sent to the regional reference laboratory, located in another state, Amazonas (LACEN-AM). Additionally, these tests are not always available at the regional laboratory, and samples must then be forwarded to the National reference laboratory in Rio de Janeiro. This process can significantly increase the time required to obtain diagnostic results and decrease the chances of obtaining viable bacteria. Furthermore, the phenotypic drug pDST panel utilized between November 2021 and December 2022 did not encompass bedaquiline or linezolid.

The high rate of absence of second-line testing absence is expected to be reduced with the recent expansion of the second-line LPA (MTBDRsl) into the routine of LACEN laboratories (da Brasil, 2023), decreasing as such the need to send samples to the regional or national reference center and accelerating the diagnostic process. However, it is crucial to note that LPA has limitations, such as the lack of detection of mutations outside the regions targeted by the probes, and the impact of bacillary load on the test performance (Barnard et al., 2020; World Health Organization and Others, 2022). Moreover, studies have shown that LPA failed to detect isoniazid resistance in 33% of isolates due to the presence of low resistance mutations in *katG, inhA*, and the *inhA* promoter (Brossier et al., 2006), or to detect rifampicin resistance on sputum samples with negative smear compared to the Xpert MTB/RIF (Yadav et al., 2021).

In this context, it is important to perform minimum inhibitory concentration (MIC) for second-line and new drugs in at least some laboratory routines in each of the five main Brazilian regions, due to LPA limitations. The performance of MIC assay will allow the detection of resistance to the new drugs and also, to detect resistances, that uncovered by LPA providing important information for the description of and also unknown patterns of resistance, only patterns, normally detected by phenotypic testing methods that could be further investigated and elucidated by WGS (Nimmo et al., 2024).

Other discrepancies between WGS and SOC were mainly associated with low-frequency variants related to the development of resistance in subpopulations of the main strain and the evolution of the resistance pattern within the host. Detecting those variants is an important step in the MAGMA pipeline, allowing us to detect sub-populations within the main strain (Heupink et al., 2021, 2023). This phenomenon is often associated with happening during the emergence of resistance; those small populations and such subpopulations might occur in a frequency even below the detection threshold of culture-based methods (Cohen et al., 2019).

Accurate variant calling and critical interpretation are essential for understanding the link between mutations and drug resistance, particularly for low-frequency variants. These variants, often associated with small resistant populations or sequencing errors, may not currently correlate with phenotypic resistance. However, a recent study (de Vos et al., 2019) highlights the potential for low-frequency variants to become dominant under antibiotic pressure, as seen in patients with treatment lost to follow-up or relapse. For patients treated with bedaquiline-based therapies, especially those lost to follow-up (like IEC0264 with detection of low frequency of resistance-related mutation), WGS is crucial to assess the acquisition of additional variants and to formulate a treatment scheme. In our study, we proposed to disregard low-frequency mutations that occur in less than 3% of the population, such as the *mmpR5* c.386delC variant (0.02) that occurred in the strain IEC1485, where a person completed bedaquiline-based treatment with a positive treatment outcome of cure.

Finally, the third category of discrepancies was associated with mutations of unknown association to resistance by WHO, as observed in three strains: IEC1485, IEC0614, and IEC1712, with phenotypic resistance to isoniazid. Further studies are needed to investigate the impact of these variants on isoniazid resistance development. These variants were *katG*_c.1349_1351delTCG, *katG*_p.Ala621Ser, *katG*_c.-683 T > A, *glpK*_c.571dupG, *katG*_c.-343G > T, and *katG*_c.-697 T > A, which should be confirmed with Sanger sequencing and MIC and analyzed by protein modeling.

The results from WGS provide critical insights that extend beyond the scope of SOC diagnostics. While SOC primarily focuses on first-line drugs and selected second-line agents, WGS offers a more comprehensive drug resistance profile, including the detection of mutations associated with newer antimicrobials such as bedaquiline, linezolid, clofazimine, and delamanid. In this study, WGS identified low-frequency variants linked to resistance against these drugs, which were not detectable through SOC testing. For instance, bedaquiline resistance mutations were detected in multiple strains, underscoring the importance of WGS in identifying emerging resistance patterns that are critical for optimizing treatment regimens. Furthermore, WGS can detect resistance mutations for drugs not routinely tested in SOC workflows due to logistical or technical limitations, providing clinicians with a broader understanding of potential therapeutic options. This enhanced diagnostic capability of WGS ensures a more tailored treatment approach, ultimately improving patient outcomes and aiding in the containment of DR-TB transmission. These findings highlight the indispensable role of WGS in complementing SOC diagnostics and underscore its potential to revolutionize the management of DR-TB in high-burden settings.

Our findings highlight the urgent need for enhanced genomic surveillance and targeted public health interventions in Pará. The emergence of XDR-TB strains, particularly in urban centers like Belém, poses a significant challenge to TB control efforts. The use of WGS as a routine diagnostic tool could provide valuable insights into resistance mechanisms and transmission pathways, enabling more effective and tailored treatment regimens (Nimmo et al., 2024).

Real-time WGS enables the rapid detection of mutations linked to transmission chain reconstruction, aiding decision-makers in monitoring, understanding, and preventing the spread of bedaquiline-resistant and other new drug-resistant strains (Larsson et al., 2024). Investing in WGS-based surveillance, with a focus on cost reduction and effective long-term analysis, can help maintain robust drug regimens such as bedaquiline–pretomanid–linezolid–moxifloxacin (BPaLM) (WHO, 2022). The costs of WGS should be weighed against the significant investment required for new drug discovery, which ranges from $161 million to $4.54 billion (Schlander et al., 2021). Implementing WGS for diagnostics and surveillance in Brazil could particularly help reduce the lengthy delays in second-line testing, facilitating the prediction of resistance and transmission chains. Additionally, we suggest that future microbiome research should consider analyzing samples without NALC/NAOH decontamination to better understand the role of non-mycobacterial infections, including viruses other than HIV, in patient outcomes. Our current study used the standard diagnostic flow, and all patients were HIV-negative.

While this study offers valuable insights into XDR-TB emergence and transmission, its reliance on LPA, MGIT, and proportions methods, without MIC testing, especially for second-line drugs including bedaquiline and linezolid, limits the generalizability of the comparison to WGS analyzed with the WHO catalog. To gain a more comprehensive understanding of XDR-TB transmission dynamics in Brazil, future studies should be performed, increasing not only the sample size, including a broader geographic period but also the study area, and conducting longitudinal analyses to better assess the impact of bedaquiline and other new drugs on the resistance the emergence of DR in the country. In Brazil’s current diagnostic workflow, sputum samples are decontaminated and cultured in Ogawa Kudoh media at frontier laboratories, with positive cultures sent to state LACENs for further analysis. Implementing real-time *M. tuberculosis* WGS at each of Brazil’s 27 state LACENs could significantly enhance genomic surveillance, leveraging existing WGS infrastructure from the COVID-19 pandemic.

## 5 Conclusion

This study provides important insights into the emergence, transmission dynamics, genetic diversity, and resistance profiles of the first XDR-TB cases in Pará, Brazil, within the cohort of RR/MDR-TB patients treated with bedaquiline-based regimens. By integrating SOC and WGS analyses, we identified multiple transmission clusters, including mixed infections with different *M. tuberculosis* lineages and co-infections with NTM. We demonstrated the importance of genomic surveillance in detecting and monitoring emerging drug-resistant TB strains, especially in high-burden areas such as Belém. Identifying transmission chains associated, with an example being the evolution of the local prevalent strain with SIT 2517 to pre-XDR and XDR belonging to SIT 2517 (an endemic SIT isolated only in Pará) enforces the importance of continuous genomic surveillance to identify the emergence of resistance and its spread. Showcasing the use of WGS in the detection of resistance-associated variants and transmission chains, particularly in resource-limited settings like Pará, where phenotypic testing for new drugs remains limited. The discrepancies between SOC and WGS results, almost surely due to the absence of second-line testing, show critical gaps in current diagnostic protocols that could be reduced by the implementation of WGS in laboratory routines. The implementation of WGS as a routine tool could mitigate these delays, providing timely insights into resistance mechanisms and enabling more effective regimen prescriptions. The interpretation of minor variants detection (low frequency) should be further investigated and carefully performed to guide patient care.

## Data Availability

The datasets presented in this study can be found in online repository NCBI under the Bioproject ID PRJNA1135144. The names of the accession number(s) can be found in the article/Supplementary material.

## Footnotes

^1^ http://www.ial.sp.gov.br/resources/insituto-adolfo-lutz/centros-tecnicos/centro-de-bacteriologia/report_resistencia_mtb.pdf

^2^ http://gal.saude.pa.gov.br/

^3^ http://sitetb.saude.gov.br/

^4^ https://github.com/TORCH-Consortium/MAGMA/releases/tag/v2.0.0

^5^ https://itol.embl.de/

^6^ https://github.com/xiaeryu/SpoTyping-v2.0

^7^ http://www.pasteur-guadeloupe.fr:8081/SITVIT2

^8^ https://www.arcgis.com/
